# Carriage of Extended-Spectrum Beta-Lactamase-Plasmids Does Not Reduce Fitness but Enhances Virulence in Some Strains of Pandemic *E. coli* Lineages

**DOI:** 10.3389/fmicb.2016.00336

**Published:** 2016-03-17

**Authors:** Katharina Schaufler, Torsten Semmler, Derek J. Pickard, María de Toro, Fernando de la Cruz, Lothar H. Wieler, Christa Ewers, Sebastian Guenther

**Affiliations:** ^1^Veterinary Faculty, Institute of Microbiology and Epizootics, Freie Universität BerlinBerlin, Germany; ^2^Robert Koch InstituteBerlin, Germany; ^3^Wellcome Trust Sanger InstituteCambridge, UK; ^4^Departamento de Biología Molecular, Instituto de Biomedicina y Biotecnología de Cantabria (UC-SODERCAN-CSIC), Universidad de CantabriaSantander, Spain; ^5^Veterinary Faculty, Institute of Hygiene and Infectious Diseases of Animals, Justus-Liebig-Universität GiessenGiessen, Germany

**Keywords:** ESBL-producing *E. coli*, ESBL-plasmids, fitness costs, enhanced virulence, biofilm formation, plasmid and host interaction

## Abstract

Pathogenic ESBL-producing *E. coli* lineages occur frequently worldwide, not only in a human health context but in animals and the environment, also in settings with low antimicrobial pressures. This study investigated the fitness costs of ESBL-plasmids and their influence on chromosomally encoded features associated with virulence, such as those involved in the planktonic and sessile behaviors of ST131 and ST648 *E. coli*. ESBL-plasmid-carrying wild-type *E. coli* strains, their corresponding ESBL-plasmid-“cured” variants (PCV), and complementary ESBL-carrying transformants were comparatively analyzed using growth curves, Omnilog® phenotype microarray (PM) assays, macrocolony and biofilm formation, swimming motility, and RNA sequence analysis. Growth curves and PM results pointed toward similar growth and metabolic behaviors among the strains. Phenotypic differences in some strains were detected, including enhanced curli fimbriae and/or cellulose production as well as a reduced swimming capacity of some ESBL-carrying strains, as compared to their respective PCVs. RNA sequencing mostly confirmed the phenotypic results, suggesting that the chromosomally encoded *csgD* pathway is a key factor involved. These results contradict the hypothesis that ESBL-plasmid-carriage leads to a fitness loss in ESBL-carrying strains. Instead, the results indicate an influence of some ESBL-plasmids on chromosomally encoded features associated with virulence in some *E. coli* strains. In conclusion, apart from antibiotic resistance selective advantages, ESBL-plasmid-carriage may also lead to enhanced virulence or adaption to specific habitats in some strains of pandemic ESBL-producing *E. coli* lineages.

## Introduction

The global emergence of antimicrobial resistance, including extended-spectrum beta-lactamases (ESBL), is driven not only by plasmids encoding for these factors, but is also crucially influenced by pandemic bacterial clonal lineages (Naseer and Sundsfjord, 2011). The success of the pathogenic ESBL-producing *E. coli* clonal lineage of sequence type ST131 and virulence-associated phylogenetic group B2 is particularly noteworthy (Nicolas-Chanoine et al., 2014). B2-ST131 is found worldwide in environments with high antimicrobial selection pressures, including human and veterinary clinics and communities (Nicolas-Chanoine et al., 2008; Ewers et al., 2010). ST131 is also found in remote areas and wildlife (Bonnedahl et al., 2014), where antimicrobial influence is thought to be of lower importance. Despite the recognition of the human clinical reservoir as most abundant, several studies have demonstrated increasing prevalence of ST131 in animals and extra-clinical settings (Ewers et al., 2012). The acquisition of ESBL-genes happened over time, and the initial spread of ST131 most likely evolved from an emergence of chromosomally encoded fluoroquinolone resistance (Nicolas-Chanoine et al., 2014). Besides ST131, several STs, including ST648, ST617, ST167, ST410, ST224, and ST117, appear to be associated with ESBL-production, which demonstrates that ESBL-producing isolates are not equally distributed over all phylogenetic backgrounds (Ewers et al., 2012). This is expected in cases of solely plasmid-driven spread (Ewers et al., 2012). Regarding the success of pathogenic clonal lineages of B2-ST131, similar scenarios might also apply to CTX-M-producing lineages of ST648 belonging to phylogenetic group D, also known for harboring virulent isolates (Pitout, 2012; Ewers et al., 2014). Many strains of ST131 and ST648 carry plasmids encoding ESBL-enzymes, often of the CTX-M-15 type, and have become problematic due to limitations in antimicrobial therapies (Johnson et al., 2010). Besides ESBL-encoding genes, plasmids of these *E. coli* isolates contain antibiotic resistance determinants affecting various antimicrobial classes, which often results in multi-drug resistant phenotypes (Woodford et al., 2011). Prior studies suggest trade-offs between antibiotic-resistance and fitness in such strains (Dasilva and Bailey, 1986; Lenski, 1997; Andersson and Hughes, 2010). However, this does not necessarily apply to ST131 and ST648. In contrast, the combination of multi-resistance, virulence and phylogenetic background is hypothesized to be a recipe for their successful pandemic spread (Johnson et al., 2010; Pitout, 2012; Calhau et al., 2013). In addition to antibiotic resistance genes, ESBL-plasmids harbor non-resistance factors, which are partly unexplored. These include fertility and virulence factors, genes for plasmid maintenance including toxin-antitoxin systems, resistances against heavy metals (Seiler and Berendonk, 2012; Schaufler et al., 2013), and putative protein-coding genes (Smet et al., 2010b).

Why do ESBL-associated STs exist, and why are certain clonal lineages so successful not only in environments with high and moderate antibiotic pressures, but also in antimicrobially isolated areas? It might be due to their ubiquity and frequent detection, whereas rare lineages are found less often, or for reasons beyond antibiotic resistance such as virulence-associated factors. One possibility has rarely been studied: the interaction between ESBL-plasmids and the chromosomal content of particular clonal lineages. The influence of non-resistance genes on the chromosome may be of particular importance. For the closely related species *Klebsiella pneumoniae*, it was shown that acquisition of ESBL-plasmids lead to expression changes of chromosomally encoded fimbriae genes, subsequently affecting the overall invasion ability of tested strains (Sahly et al., 2008). Another main bacterial virulence factor is the chromosomally encoded ability to form biofilms. The subtle interactions between biofilm formation and its counterpart, motility, are mostly regulated by the transcriptional regulator *csgD* (curlin subunit gene D; Hammar et al., 1995; Dudin et al., 2014). Biofilm formation has previously been linked to antimicrobial resistance (Ito et al., 2009); however, the influence of ESBL-plasmids on *csgD-*associated virulence features remains to be investigated.

This study addressed two hypotheses: (i) ESBL-plasmid carriage does not negatively influence the host's growth/metabolic fitness; and (ii) ESBL-plasmid carriage supports the host through the ESBL-plasmid's influence on chromosomally encoded virulence-associated features.

## Materials and methods

### Strains

Seven wild-type (WT) ESBL-producing *E. coli* strains, their ESBL-“plasmid” cured variants (PCV) (Schaufler et al., 2013), and transformants (T) with the reintroduced large ESBL-plasmid constructed from PCVs (Green and Sambrook, 2012) were analyzed in this study; in total, 21 strains were studied. The WT B2-ST131 and D-ST648 strains originated from different hosts including humans, companion animals, and wild birds (Supplementary Table S1).

### ESBL-plasmid-“curing” and transformations

As previously described by Schaufler et al. (2013), large ESBL-plasmids were extracted from seven WT ESBL-carrying *E. coli* strains using a heat technique (Dale and Park, 2004). Loss of the large ESBL-plasmid as well as the clonal character of WT and its corresponding PCV strain were tested via plasmid-profile analysis and *Xba*I-pulsed-field gel electrophoresis (PFGE) (Schierack et al., 2009). To assure genetic identity and to exclude chromosomal changes in the PCV, the whole genome sequences of WT and PCV strains were analyzed using bioinformatics. This included a pairwise comparison of the number of orthologous genes using the OrthoMCL pipeline (Chen et al., 2006) and computation of the phylogenetic distances of all strains based on their Maximum Common Genome (MCG) (Von Mentzer et al., 2014) as previously described (Schaufler et al., 2013). Transformants with the reintroduced large ESBL-plasmid were constructed to verify the observed phenotypic differences (Supplementary Table S1). PCVs functioned as electrocompetent acceptor strains; thus, the respective large ESBL-plasmid was transformed via electroporation (Green and Sambrook, 2012). Transformed strains were screened for ESBL-production on cefotaxime (4 μg/ mL; Sigma-Aldrich, Taufkirchen, Germany) containing CHROMagar™ (MAST Diagnostica, Reinfeld, Germany) plates for ESBL-enzyme-production. Plasmid profile analysis was used to evaluate the success of electroporation.

### ESBL-plasmid characteristics

**Figure 1:**
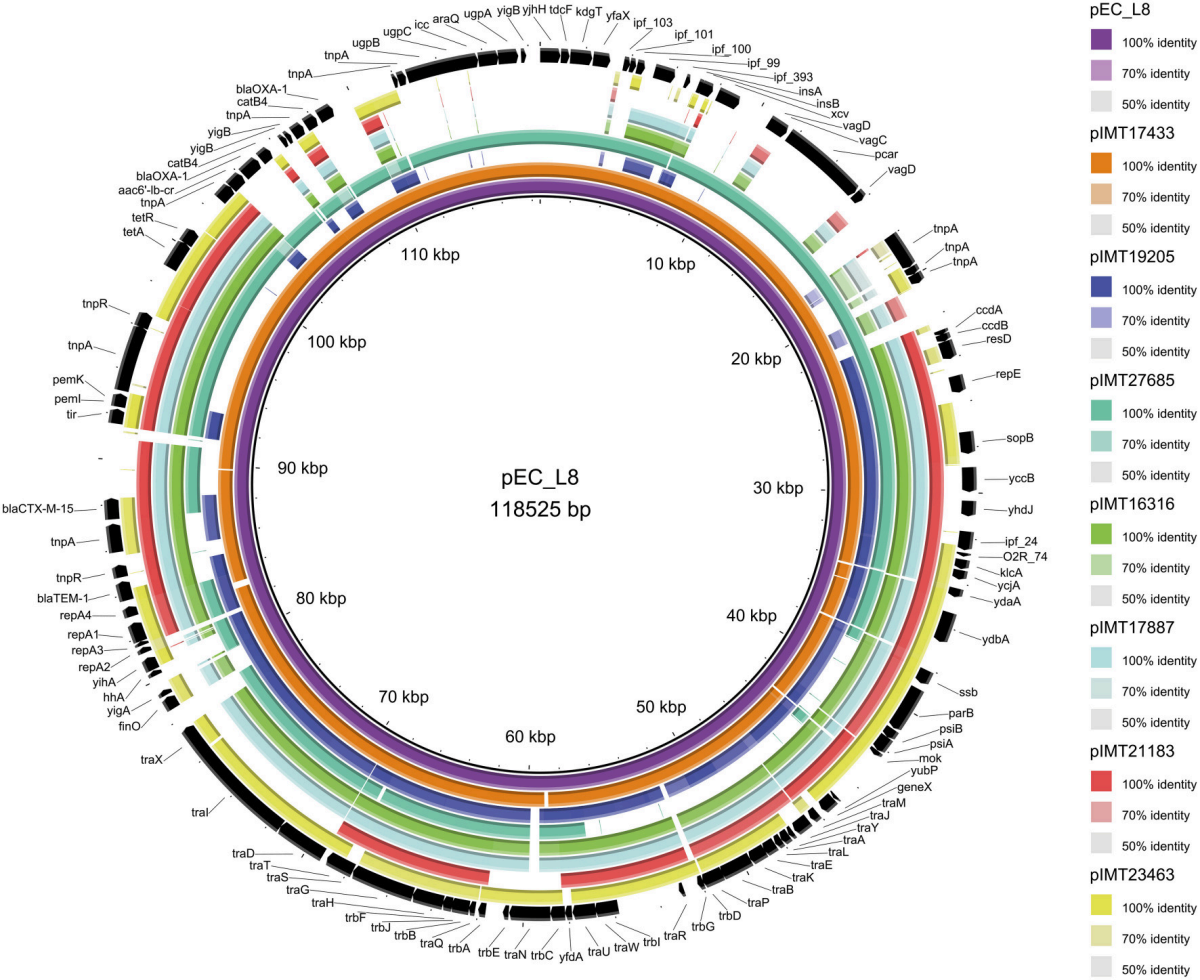
**Circular visualization of the seven wild-type ESBL-plasmid sequences as compared to ***E. coli*** ESBL-plasmid pEC_L8 using BRIG (Alikhan et al., 2011)**.

Whole genomes of all WT and their corresponding PCV strains were sequenced using an Illumina HiSeq2000 sequencer in collaboration with the Wellcome Trust Sanger Institute (Cambridge, United Kingdom). The resulting reads were used for a *de novo* assembly (CLC Genomics Workbench 6.5, CLC Bio, Denmark). PLACNET analysis (Lanza et al., 2014) was then performed for all genomes to extract putative plasmid contiguous sequences (contigs) and to assign them to the large ESBL-plasmid and to smaller, additional non-ESBL-plasmids. These were confirmed via BLAST analysis, in which they were compared to reference sequences of known plasmids. All previously defined plasmid contigs showed a high similarity to parts of plasmids that have already been described. Annotation of the contigs was performed using the annotation feature of the program Geneious version 7.1.2 (Kearse et al., 2012) with 100% similarity to an in-house plasmid reference data base (data not shown). ESBL types, relaxase (REL) and plasmid replication initiator (RIP) proteins, incompatibility (Inc) groups, approximate ESBL-plasmid sizes, the number of additional plasmids, and virulence factors were detected based on PLACNET ESBL-plasmid sequences and using VirulenceFinder 1.4 (Joensen et al., 2014) and ResFinder 2.1 (Zankari et al., 2012; Table 1). BLAST ring image generator (BRIG) (Alikhan et al., 2011) was used to visualize the annotated PLACNET ESBL-plasmid sequences (pIMT17433, pIMT19205, pIMT27685, pIMT16316, pIMT17887, pIMT21183, and pIMT23463) of the seven WT strains with pEC_L8 as a reference ESBL-plasmid (Smet et al., 2010b; Figure 1).

**Table 1:** **Characteristics of large ESBL-plasmids of the seven ESBL-producing wild-type strains**.

**Strain**	**ESBL type**	**ESBL-plasmid REL/RIP proteins and Inc group**	**ESBL-plasmid size (bp)**	**Number of additional plasmids (sizes in bp)**	**ESBL-plasmid additional resistance genes**	**ESBL-plasmid virulence genes**
IMT17433	CTX-M-15	MOB_F12_, RepFIA/FII, IncF	122,823	1 (1600)	*bla*_TEM−1_, *bla*_OXA−1_, *tet(A), tet(R), aadA, aac(6′)-Ib-cr, catB4*	*finO, traT*
IMT19205	CTX-M-27	MOB_F12_, RepFIA/FIB/FII, IncF	166,151	2 (1500, 5200)	*bla*_TEM−1_*, tet(A), sul2, strA, aadA, aac(3)-IId, aac(3)-IV, aac(6′)-Ib-cr*	*finO, traT, senB*
IMT27685	CTX-M-15	MOB_F12_, RepFIA/FII, IncF	133,611	1 (1600)	*bla*_OXA−1_*, tet(A), sul1, strA, strB, aadA, aac(6′)-Ib-cr, dfrA17*	*traT*
IMT16316	CTX-M-15	MOB_F12_, RepFIA/FIB, IncF	136,508	4 (2500, 3200, 4100, 7000)	*bla*_TEM−1_*, tet(A), tet(R), sul1, sul2, strA, strB, aadA, aac(3)-II, dhfrVII, dfrA17*	*finO, traT*
IMT17887	CTX-M-15	MOB_F12_, RepFIA/FIB, IncF	143,494	2 (2600, 7100)	*bla*_TEM−1_*, tet(A), tet(R), sul1, sul2, strA, strB, aadA, aac(3)-IId, aph(3′)-Ia, dhfrVII, dfrA17*	*finO, traT*
IMT21183	CTX-M-15	delta-TraI, RepFIA, IncF	103,420	1 (7100)	*bla*_TEM−_1*, tet(A), tet(R), sul1, sul2, strA, strB, aadA, aac(3)-II, aph(3′)-Ia, dhfrVII, dfrA17*	*traT*
IMT23463	CTX-M-14	MOB_F12_, RepFIB/FII, IncF	143,193	1 (4100)	*bla*_TEM−1_*, bla*_OXA−1_*, tet(A), sul2, strA, strB, aadA, aac(3)-IId, aac(6′)-Ib-cr, dfrA17*	*finO, traT, sitABCD, cma*

*REL, relaxase; RIP, replication initiator protein; Inc, incompatibility; bp, base pairs*.

### Growth curves

Growth curves in LB medium were performed in triplicate using standard protocols.

### Omnilog® phenotype microarray (PM)

Using 96-well microtiter plates spotted with different substrates, growth/metabolic activity in 379 single substrates and sensitivity to 48 antimicrobial and chemical compounds was analyzed for all strains [Omnilog® (Biolog, Hayward, USA), Supplementary Table S2]. Three biological replicates were tested on six PM plates (PM1 and PM2: carbon sources; PM3: nitrogen sources; PM4: phosphorus and sulfur sources [http://www.biolog.com/pdf/pm_lit/PM1-PM10.pdf]; PM13 and PM14: chemical sensitivity [http://www.biolog.com/pdf/pm_lit/PM11-PM20.pdf]). Plates were inoculated with the bacterial suspension according to Omnilog® PM protocols. Plates were incubated for 48 h at 37°C. Respiration was measured by dye (tetrazolium violet) reduction every 15 min. The calculated longitudinal respiration kinetics were analyzed in R (Vaas et al., 2013). In confidence interval plots computed with extract defined by enlisted metadata for the parameter “area under the curve,” only wells in which the normalized mean point estimates of WT and T groups showed no overlapping 95% confidence intervals as compared to those of the PCV group, were considered to be significantly different. The group means of these wells underwent further statistical analysis using Tukey's method for multiple comparisons.

### Macrocolonies

Three microliters of overnight culture (from a single colony grown in 5 mL BHI broth) from all strains were dropped on span agar plates (H. Carroux, Germany) with or without sodium chloride (5%) and congo red solution [0.5% congo red (Sigma-Aldrich, Taufkirchen, Germany) and 0.25% coomassie-brilliant-blue (Carl Roth, Karlsruhe, Germany) diluted in ethanol]. Plates were incubated for 5 days at 28°C (Romling, 2005; Richter, 2011). Following an initial comprehensive screening to detect differences between WT and PCV strains regarding their cellulose and/or curli fimbriae production, follow-up runs were performed on plates containing and lacking sodium chloride for all strains (Table 2). These follow-up runs were repeated six times. Reference strains (AAEC189, Blomfield et al., 1991, IMT26949, and W3110 Hayashi et al., 2006) were included in all runs for all plates and at all temperatures.

**Table 2:** **Cellulose and curli fimbriae expression by 17433 and 16316 strains on span agar plates with congo red, with or without sodium chloride, incubated at 28°C for 5 days**.

**Strain**	**With sodium chloride**		**Without sodium chloride**	
	**Cellulose**	**Curli**	**Cellulose**	**Curli**
IMT17433	^******^	^******^	^******^	^******^
PCV17433				
T17433	^*****^	^******^	^*****^	^******^
IMT16316	^******^	^******^		
PCV16316		^******^		
T16316	^******^	^******^		

Six repetition runs followed a comprehensive initial screening. ^*^, positive cellulose and/or curli fimbriae phenotype per run; 16316 strains were not tested on plates lacking sodium chloride as they did not show any differences during the initial screening.

### Biofilm formation assays

Overnight culture (from a single colony grown in 5 mL LB and minimal medium M63 overnight, Pardee et al., 1959) was set to optical density OD_600_ = 0.05. Technical triplicates of the suspension were added to a 96-well plate, which was then hermetically closed and incubated for 24 or 48 h at 28, 37, or 42°C. Reference strains (AAEC189 and W3110) were included in all runs in triplicate for each medium, at all temperatures and time points. After incubation, OD was measured with an ELISA-reader (Synergy HT, BioTEK Instruments, Bad Friedrichshall, Germany) and bacteria were washed with aqua bidestillata, fixed in 99% methanol, stained with 0.1% crystal violet, and dissolved in 80:20 ethanol:acetone. After dissolution, OD was again measured, then biofilm formation capacity was computed (Martinez-Medina et al., 2009) and statistically analyzed using IBM SPSS Statistics for Windows, Version 20 (Dunn, 2013). Normal distributions of measuring points of all groups (strains) were tested using Kolmogorov-Smirnov (Smirnov, 1948). Based on the non-normal distributions of the measuring points of all groups, the non-parametric Wilcoxon rank-sum test (Mann-Whitney *U*-test; Wilcoxon, 1945) was used to estimate whether the observed biofilm formation capacity differences were statistically significant (*p* = 0.05). This assay was repeated three times in triplicate per temperature, and in two media for all strains.

### Motility assays

Overnight culture (from a single colony grown in 5 mL BHI broth) was set to OD_600_ = 1. One milliliter was centrifuged and washed twice with 1 × phosphate buffered saline (PBS). Five microliters of the suspension were dropped onto swim-agar plates (LB and 0.3% agar). Plates were incubated at 28, 37, and 42°C. The strain MG1655 (Hayashi et al., 2006; Richter, 2011) was used as a control for a positive swimming phenotype. After 48 h, colony diameters were measured (Harshey, 2003) and statistically analyzed using IBM SPSS Statistics for Windows, Version 20 (Dunn, 2013). Normal distributions of measuring points of all groups (strains) were tested using Kolmogorov-Smirnov (Smirnov, 1948). Based on the non-normal distributions of the measuring points of all groups, the non-parametric Wilcoxon rank-sum test (Wilcoxon, 1945) was used to estimate whether the observed swimming differences were statistically significant. The significance level for multiple comparisons between WT, PCV and T groups was adjusted to *p* = 0.016. This assay was repeated six times.

### RNA sequencing

The RNA of IMT17433, PCV17433, and T17433 was sequenced. RNA was isolated from two biological replicates from each of two macrocolony and two motility plates. RNA was isolated from cells using the RNASnap method (Stead et al., 2012) and shipped to LGC Genomics (Berlin, Germany) for RNA sequencing with an Illumina HiSeq2000 producing one channel paired end reads. The details of the company's standard protocols for quality control, RNA extraction from the RNASnap technique and rRNA depletion (using Ribo-Zero (Epicentre), Biozym, Hessisch Oldendorf, Germany) can be found on their website (http://www.lgcgroup.com/services). cDNA synthesis, library generation, indexing and cluster generation were performed using Illumina technology (TruSeq RNA Sample Preparation Kit v2). Bioinformatic mRNA differential expression analysis included the following processing steps: (a) generating FastQC reports to check the quality of sequenced reads; (b) clipping Illumina TruSeq sequencing adapters from the 3′ ends of reads; (c) filtering rRNA reads using riboPicker (Schmieder et al., 2012); (d) aligning reads against IMT17433 as a reference using TopHat2 (Kim et al., 2013) and RSEM; (e) counting reads per gene using HTSeq-count, and per transcript and gene using RSEM (Li and Dewey, 2011); and (f) computing differential gene and transcript expression between different groups of samples (including technical and biological replicates) using R/Bioconductor packages DESeq (Anders and Huber, 2010), edgeR (Robinson et al., 2010), and Cuffdiff (part of the Cufflinks software package, Trapnell et al., 2013).

## Results

### ESBL-plasmid-“curing” and transformations

All PCVs kept their smaller plasmids and lost only the large ESBL-plasmid. Bioinformatic analysis of all WT and PCV genomes assured their genetic similarity, ruling out any changes in the PCVs' chromosomal content during the “curing” procedure (Schaufler et al., 2013; Von Mentzer et al., 2014). To verify detected phenotypic differences between WT and PCV strains, seven transformants containing the reintroduced large ESBL-plasmid were used (T17433, T19205, T27685, T16316, T17887, T21183, and T23463; Supplementary Table S1). These strains showed phenotypic cefotaxime-resistance (CLSI, 2008). Plasmid-profile analysis confirmed transformation of the large ESBL-plasmid.

### ESBL-plasmid characteristics

Whole genomes of all WT strains were used for plasmid characterization (http://www.sanger.ac.uk/resources/downloads/bacteria/escherichia-coli.html#project_2119; IMT17433 [ERR163891], IMT19205 [ERR163889], IMT16316 [ERR163879], IMT17887 [ERR163883], IMT21183 [ERR163880], IMT23463 [ERR163881]; http://www.sanger.ac.uk/resources/downloads/bacteria/escherichia-coli.html#project2433; IMT27685 [ERR264283]). Table 1 summarizes the most important ESBL-plasmid characteristics. Their sizes ranged from approximately 100–166 kb. Besides common *bla*_CTX−M_ types (mostly the CTX-M-15 enzyme), non-beta-lactam resistance genes (e.g., *tet(A)/(R)* and *aac(6*′*)-Ib-cr*) were found. Virulence-associated genes, mainly *finO* and *traT*, were present. All WT strains harbored at least one smaller non-ESBL-plasmid (Table 1). Analysis of PCV genomes showed complete loss of the MOB_F12_/IncF ESBL-plasmids in six of the seven genomes analyzed. Strain IMT19205 contained two IncF plasmids that were unresolvable by PLACNET. This strain lost its ESBL-phenotype along with one of the IncF plasmids.

Figure 1 displays the annotated ESBL-plasmid sequences (pIMT17433, pIMT19205, pIMT27685, pIMT16316, pIMT17887, pIMT21183, and pIMT23463) as compared to ESBL-plasmid pEC_L8 (Smet et al., 2010a), showing both beta-lactam and non-beta-lactam resistance genes, as well as non-resistance genes, including *icc* (phosphodiesterase) and *hha* (hemolysin expression modulating protein), which were present on all plasmids. Additionally, this comparison displays typical features of conjugative plasmids (*tra* regions and insertions sites) and plasmid partitioning (toxin-antitoxin systems: e.g., *vagC/D* and *pemI/K*).

In summary, although they contained similar genetic backbones and resistance determinants, ESBL-plasmids were rather diverse.

### Growth curves and Omnilog® phenotype microarray (PM)

No differences were detected between the LB growth curves of WT ESBL-producing *E. coli* strains and the corresponding PCVs (data not shown).

Growth/metabolic activity and chemical sensitivity of all strains were then screened using the Omnilog® PM system. Only a minor proportion of strains showed significant differences between the 427 tested compounds (Supplementary Table S2). Exemplary for plate PM1, significant differences were observed for strain combinations 17433 and 17887. Additionally, plates PM2 and PM3 revealed few significant differences (Supplementary Table S2). In contrast, on plate PM4 (sulfur and phosphorus sources), significant differences were observed for 22 wells, of which 18 showed higher values in WT and T strains, while four showed higher values in PCV strains.

The chemical sensitivity plates PM13 and PM14 revealed expected significant differences for ESBL-carrying (WT and T) strains in antimicrobial-containing wells, which showed higher metabolic values than corresponding PCV strains (e.g., PM13, C6: Doxycycline and D4: Cefuroxime; and PM14, G8: Carbenicillin; Supplementary Table S2).

In summary, given the high numbers of substrates tested, only a small proportion of wells showed significant differences in the “area under the curve” parameter between WT, PCV, and T strains. The detected significant differences were bidirectional, meaning that in some cases the PCV strains showed higher growth/metabolic activity values, and in other cases the ESBL-plasmid-carrying strains showed higher growth/metabolic activity values.

### Macrocolonies

The expression of the biofilm-associated extracellular matrix components cellulose and/or curli fimbriae was tested using macrocolony assays. Table 2 shows the results of the six follow-up runs, which were performed for those strains with differences in cellulose and/or curli fimbriae production during the initial screening (combinations 17433 and 16316).

**Figure 2:**
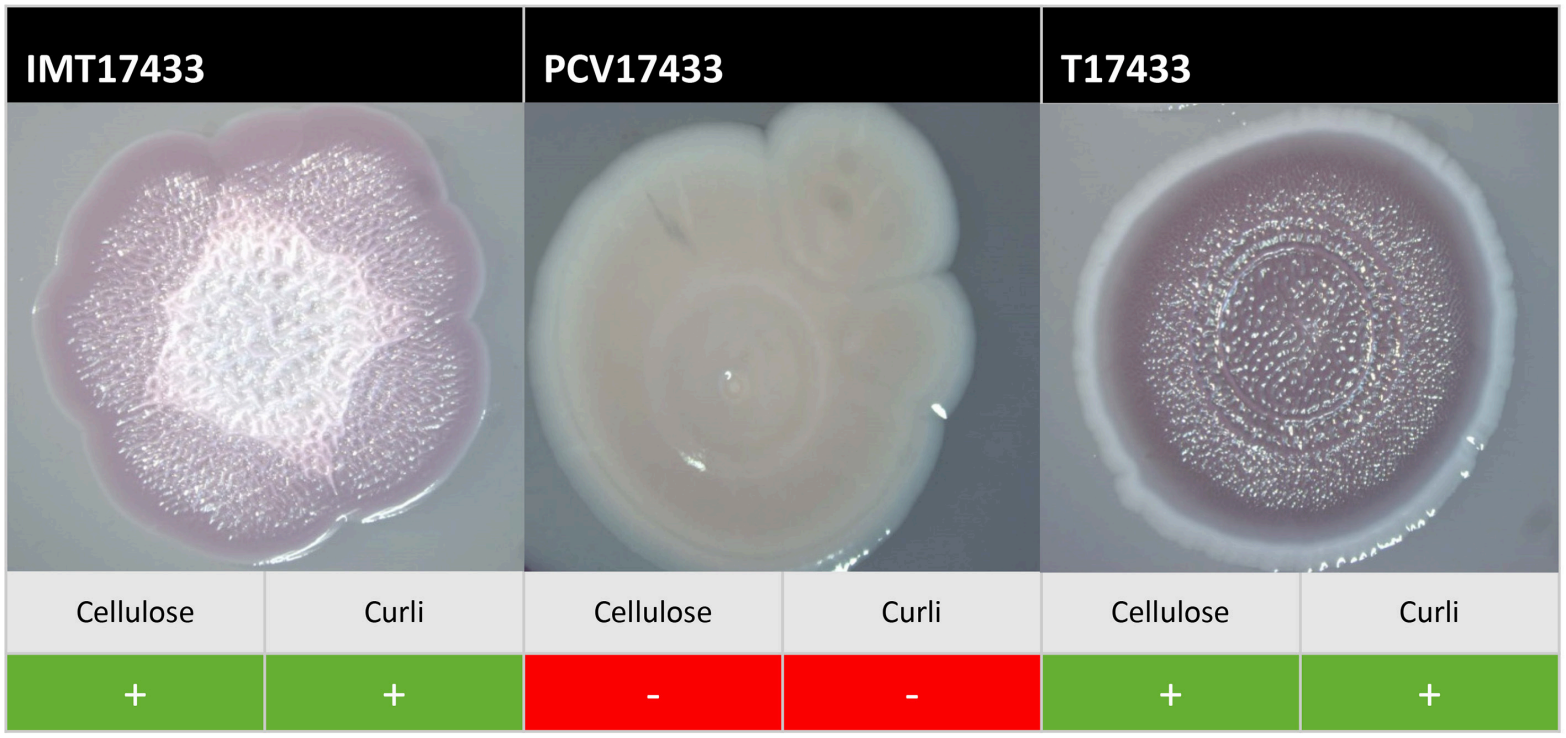
**Exemplary macrocolonies of IMT/PCV/T17433**. Span agar plates with congo red and sodium chloride, incubated for 5 days at 28°C.

On plates containing sodium chloride, ESBL-carrying WT IMT17433 expressed cellulose and curli fimbriae, whereas its corresponding PCV did not (Figure 2). Another mutant (PCV16316) did not show any cellulose expression in any run as compared to its associated WT strain (IMT16316), which produced both cellulose and curli fimbriae. In both cases, in accordance with the corresponding WT strains, the transformants T17433 (Figure 2) and T16316 produced curli fimbriae and cellulose in most runs. Only combination 17433 showed the same results on plates lacking sodium chloride (Table 2).

In summary, PCV17433 and PCV16316 displayed reduced production of extracellular components as compared to their respective wild-type and transformant strains.

### Biofilm formation assays

**Figure 3:**
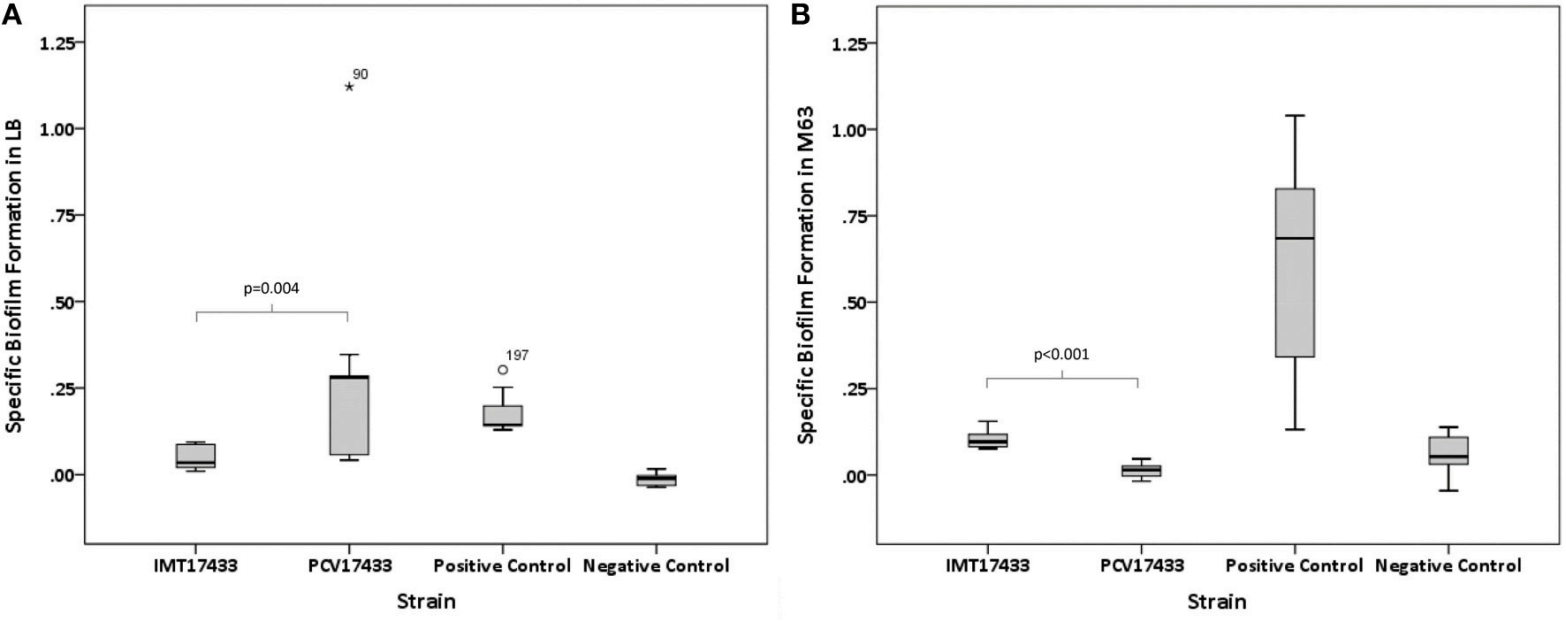
**(A)** Box plots of the distributions of the specific biofilm formation capacities of IMT17433 and PCV17433 at 37°C after 48 h in LB medium. Plots were generated using IBM SPSS Statistics for Windows, Version 20. Positive control, AAEC189; Negative control, W3110. **(B)** Box plots of the distributions of the specific biofilm formation capacities of IMT17433 and PCV17433 at 37°C after 48 h in M63 medium. Plots were generated using IBM SPSS Statistics for Windows, Version 20. Positive control, AAEC189; Negative control, W3110.

Virulence-associated biofilm assays revealed significant differences among three WT/PCV combinations (IMT/PCV17433, IMT/PCV27685, and IMT/PCV17887) after 24 h and among two combinations (IMT/PCV17433 and IMT/PCV17887) after 48 h. IMT17433 and its corresponding PCV were particularly interesting. At all three temperatures and after both time points, the WT strain showed an enhanced biofilm formation capacity in glucose-containing M63 medium (28°C, 24 h: *p* = 0.222; 37°C, 24 h: *p* = 0.008; 42°C, 24 h: *p* = 0.094; 28°C, 48 h: *p* < 0.001; 37°C, 48 h: *p* < 0.001; 42°C, 48 h: *p* = 0.008) as compared to the PCV strain. In contrast, biofilm formation by PCV17433 was better in LB medium (28°C, 24 h: *p* < 0.001; 37°C, 24 h: *p* < 0.001; 42°C, 24 h: *p* < 0.001; 28°C, 48 h: *p* = 0.006; 37°C, 48 h: *p* = 0.004; 42°C, 48 h: *p* = 0.002; Figures 3A,B). Transformants did not show any biofilm formation.

In summary, some WT and PCV combinations showed differences in their biofilm formation capacities in both directions; these results were medium-dependent.

### Motility assays

**Figure 4:**
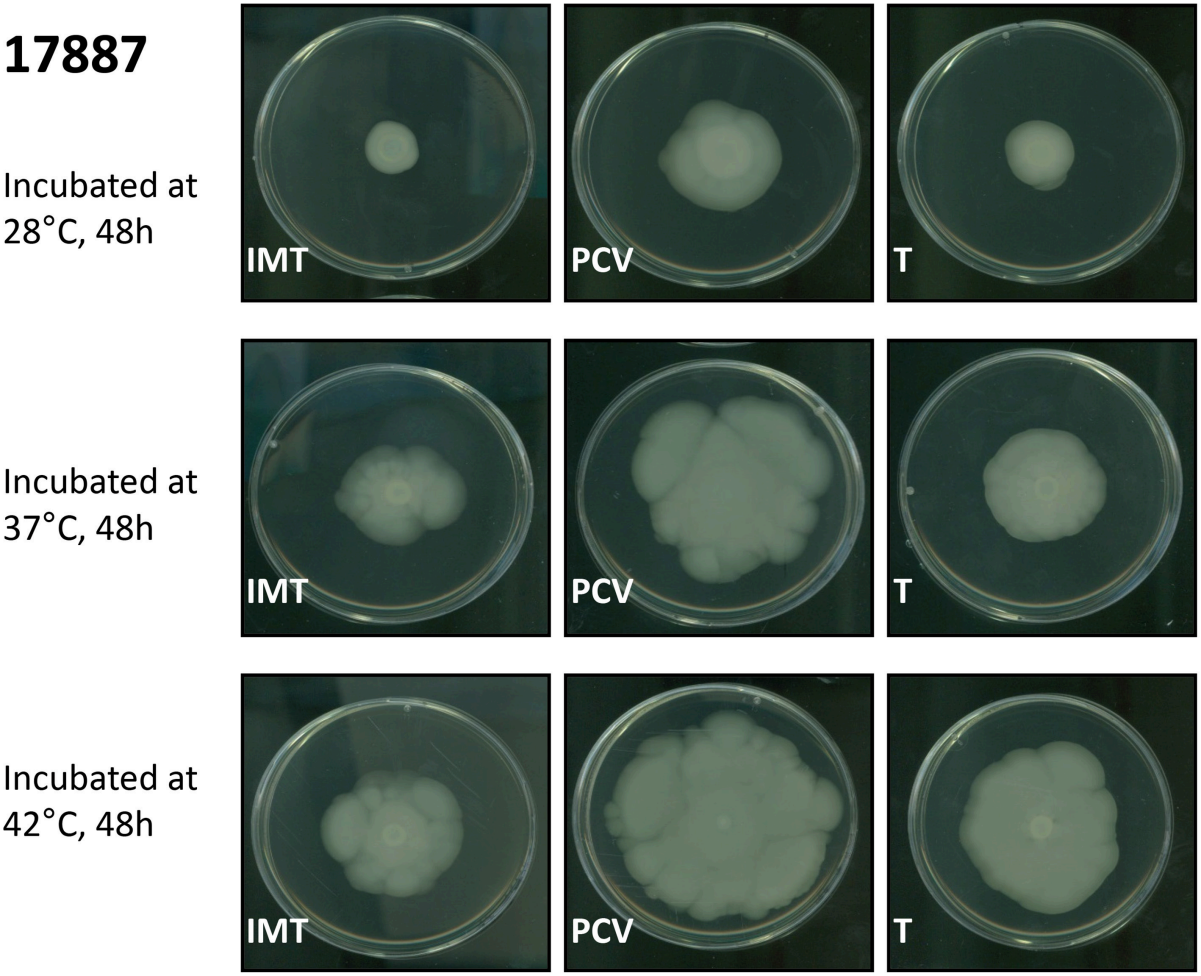
**Exemplary swimming motility of IMT/PCV/T17887**. LB plates wih 0.3% agar. Incubation at 28, 37, and 42°C for 48 h.

**Figure 5:**
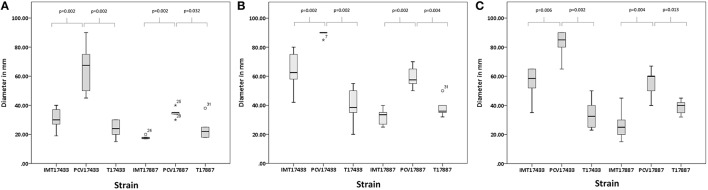
**(A)** Box plots of the distributions of the diameters, in millimetres, of the swimming capacities of IMT/PCV/T17433 and IMT/PCV/T17887 at 28°C. Plots were generated using IBM SPSS Statistics for Windows, Version 20. **(B)** Box plots of the distributions of the diameters, in millimetres, of the swimming capacities of IMT/PCV/T17433 and IMT/PCV/T17887 at 37°C. Plots were generated using IBM SPSS Statistics for Windows, Version 20. **(C)** Box plots of the distributions of the diameters, in millimetres, of the swimming capacities of IMT/PCV/T17433 and IMT/PCV/T17887 at 42°C. Plots were generated using IBM SPSS Statistics for Windows, Version 20.

Motility assays were performed to test the swimming capacity of the strains at different temperatures. Two PCV strains (PCV17433 and PCV17887) showed significantly increased swimming capacity as compared to their corresponding WT strains (IMT17433 and IMT17887) at 28, 37, and 42°C (IMT/PCV17433: 28°C, *p* = 0.002; 37°C, *p* = 0.002; 42°C, *p* = 0.006; IMT/PCV17887: 28°C, *p* = 0.002; 37°C, *p* = 0.002; 42°C, *p* = 0.004). Both transformants (T17433 and T17887) showed significantly reduced swimming capacity as compared to their corresponding PCV strains, with the exception of T17887 at 28°C. (T/PCV17433: 28°C, *p* = 0.002; 37°C, *p* = 0.002; 42°C, *p* = 0.002; T/PCV17887: 28°C, *p* = 0.032; 37°C, *p* = 0.004; 42°C, *p* = 0.013; Figures 4, 5A–C).

In summary, PCV17433 and PCV17887 showed higher swimming capacity than their respective WT and T strains.

### RNA sequencing

To gain insight into differential gene expression, RNA sequencing was performed for IMT/PCV/T17433. Only up- or downregulated genes detected with all three software packages and a threshold of 1.5 fold bidirectional regulation were considered for subsequent analysis.

Cellulose- and curli fimbriae-related differentially regulated genes from the macrocolony assay included: *csgB* (upregulation in WT compared to PCV: 9.8), *csgA* (8.6), *csgE* (6.0), *csgF* (5.5), *csgD* (5.5), *csgG* (4.0), c*sgC* (3.6), and *adrA* (2.3). Differentially regulated genes important for swimming in the motility assay were, among others: *fliZ* (downregulation in WT compared to PCV: −1.9), *flgH* (−2.0), *flgF* (−2.5), *flhC* (−2.7), *fliD* (−2.7), *flgL* (−3.0), *flgK* (−3.1), *flgC* (−3.4), *flhD* (−3.5), *fliL* (−4.0), *flgD* (−4.2), and *fliC* (−5.7). Most genes were found to be up- or downregulated in both assays, except for *csgD* and *csgC*, which were only found to be upregulated in the macrocolony assay in the WT strain as compared to the PCV strain. Conversely, downregulation of *flhC* and *flhD* was only observed in the motility assay. All cellulose- and curli-related genes that were upregulated in the macrocolony assay in IMT17433 as compared to PCV17433, were also upregulated in T17433 as compared to PCV17433. Swimming-related genes that were downregulated in the motility assay were not detected in the transformant (e.g., *fliZ, flgH, flgA, flgL, flgG, fliC*).

In summary, RNA sequencing verified the observed phenotypes for WT17433, PCV17433, and T17433 at the transcriptional level.

### Candidate genes

The observed phenotypic and transcriptomic differences were only explainable by differences at the genetic level. With only the ESBL-plasmids differing between WT/T and PCV strains and resistance determinants not explaining the results, the next step in this study was to focus on the non-resistance genes encoded by these plasmids. Table 3 shows candidate genes of the seven ESBL-plasmid sequences based on PLACNET analysis. Previous studies suggest their involvement in biofilm formation and motility. The candidate genes included *hha* (encoding the hemolysin expression modulating protein) and *yihA* (encoding a cell division protein), which were both present on pIMT17433, pIMT19205, and pIMT27685. The genes *icc* (encoding a phosphodiesterase) and *yfaX* (encoding a putative transcriptional factor) were encoded on pIMT17433 and pIMT27685. All ESBL-plasmids carried *tra* genes important for conjugation.

**Table 3:** **Candidate genes based on annotated PLACNET ESBL-plasmid sequences, literature survey, and RNA sequencing results**.

**Gene**	**Protein**	**Predicted function**	**Presence**	**References**
*hha*	Hemolysin expression modulating protein	Involved in biofilm formation and motility	pIMT17433, pIMT19205, pIMT27685	Barrios et al., 2006
*icc*	Phosphodiesterase	Involved in biofilm formation	pIMT17433, pIMT27685	Kalivoda et al., 2013
*yfaX*	Predicted DNA-binding transcriptional regulator	Putative HTH-type transcription factor	pIMT17433, pIMT27685	Perez-Rueda and Collado-Vides, 2000
*yihA*	Cell division protein, predicted checkpoint GTPase	GTP binding	pIMT17433, pIMT19205, pIMT27685	Lehoux et al., 2003
*tra*	Transfer regions of the F-conjugative plasmid	Involved in biofilm formation	all seven pIMT ESBL-plasmids	Ghigo, 2001

In summary, the detected candidate genes were mostly encoded on ESBL-plasmids of sequence type ST131 (pIMT17433, pIMT19205, and pIMT27685).

## Discussion

The success of ESBL-producing *E. coli*, particularly the pandemic pathogenic clonal lineages, cannot be explained by antimicrobial resistance alone. To assess the possibility of interactions between ESBL-plasmids and chromosomal content, we conducted a study using an unconventional approach by constructing PCVs of ESBL-plasmid-carrying WT strains of ST131 and ST648. Based on growth curves in LB medium and detailed growth/metabolic activity under different conditions, WT and PCV strains showed similar behaviors and were thus regarded suitable for analysis in subsequent phenotypic assays. Phenotypic differences between WT and PCV strains were generally verified by T strains.

In PM assays, which are a consolidation of regular LB growth curve tests (Supplementary Table S2), significant differences were detected in select wells (plates PM1-4: 3% among all wells without combination 17433). When the results of PM4 were included for 17433, 15% of the wells showed differences; however, these results need to be treated with caution since the negative control (A01) had high values for PCV17433 even after several repetitions of the assay. High values for negative controls in PM assays have previously been described for *E. coli* (Vaas et al., 2012). Differences in antimicrobial sensitivity assays on plates PM13 and PM14 between ESBL-plasmid-carrying strains and PCVs are explainable by loss of ESBL-plasmids in PCVs, which, besides ESBL-genes, carry additional antimicrobial resistance genes (Table 1; e.g., PM13, C6: Doxycycline and D4: Cefuroxime; and PM14, G8: Carbenicillin). Overall, significant differences were bidirectional, meaning that each of the ESBL-plasmid-carrying strains and the PCVs showed higher values in select wells. This indicates, as underscored by LB growth curve results, that ESBL-plasmid-carriage does not necessarily lead to fitness loss. This contradicts the hypothesis that considerable trade-offs exist between antimicrobial resistance and fitness (Dasilva and Bailey, 1986; Lenski, 1997; Andersson and Hughes, 2010). Our finding applied to all seven strain combinations irrespective of origin, ST, or ESBL-plasmid characteristics.

Biofilm-related macrocolonies result from bacterial incubation over several days, where the biosynthesis of the important virulence-related extracellular matrix components cellulose and curli fimbriae typically occurs below 30°C (Bokranz et al., 2005; Richter et al., 2014). Curli fimbriae promote adhesions to abiotic surfaces (Zogaj et al., 2001) and are associated with virulence, as they play key roles during internalization into epithelial cells (Gophna et al., 2001) and persistence in avian guts (La Ragione et al., 1999). Bacteria produce cellulose mainly as protection from both chemical and mechanical influences (Ross et al., 1991). Considering the results of the macrocolony assays for those combinations with differences, the enhanced ability of ESBL-plasmid-carrying WT and T strains to synthesize curli fimbriae and/or cellulose in contrast to their respective PCV strains was particularly notable. This indicates that ESBL-plasmid carriage confers benefits in terms of biosynthesis of virulent- and survival-associated extracellular matrix components to some bacterial strains. Several prior studies have described an influence of conjugative plasmids on biofilm formation (Ghigo, 2001; Yang et al., 2008). May and Okabe (May and Okabe, 2008) investigated the influence of natural IncF F-plasmids on biofilm formation and maturation. In this study, both conjugative (*tra* regions) and non-conjugative plasmid genes seemed to play a role. In our study, all seven ESBL-plasmids belonged to incompatibility group IncF. Furthermore, all encoded different *tra* genes are important for conjugation; however, since not all WT strains showed differences in extracellular matrix component production as compared to PCV strains, these factors are probably not solely responsible for our observed results.

Biofilm results from IMT/PCV17433 underline the complexity of biofilm formation, which is also dependent on nutrient availability. The enhanced ability of IMT17433 to form biofilms in M63 medium as compared to PCV17433 may point toward plasmid encoded features that enable the WT strain to use limited nutrients efficiently, perhaps via phosphate-dependent pathways. PM results revealing that select phosphate-containing wells in which IMT17433 showed higher respiration values than PCV17433 reinforce the latter hypothesis. Explanations of the observed enhanced ability of the PCV strain to form biofilms in LB medium, however, remain speculative. There may have been no need for planktonic IMT17433 to transform into a biofilm due to optimal utilization of the rich LB medium. A switch from a sessile (multicellular behavior, biofilm) to a planktonic (motility) way of life, and vice versa, underlies subtle interactions at a molecular level that include numerous complex cascades. Flagellar biosynthesis, for instance, seems not only crucial for bacterial swimming but also leads to surface colonization and subsequent biofilm formation. Insufficient nutrient supply leads to detachment of the cells from the biofilm and adoption of a planktonic lifestyle (Harshey, 2003).

Explanations of the observed enhanced swimming capacity among some PCV strains as compared to their corresponding WT and T strains in the motility assays remain similarly speculative. Flagellar synthesis has been shown to be energetically costly, and ESBL-plasmid-carrying strains might use their energy more strategically (Zhao et al., 2007). Alternately, due to better nutrient utilization, ESBL-plasmid-carrying strains may not have to swim as well as PCV strains to reach peripheral zones with, presumably, richer nutrition supplies.

Phenotypic differences in cellulose and curli fimbriae production as well as swimming capacity, particularly those observed between the 17433 strains, were confirmed by RNA sequencing results showing upregulation of chromosomally encoded extracellular matrix-related genes and downregulation of chromosomally encoded flagellar-related genes in IMT17433 as compared to PCV17433. Reversible RNA sequencing results for T17433 strengthen the reliability of the differences seen at phenotypic and transcriptomic levels. The key role of the transcription factor *csgD* is notable in biofilm formation and motility capacity. *csgD* regulates not only the second curli fimbriae operon *csgBAC* and *adrA*, whose product is not only accountable for several other genes involved in cellulose production, but also influences *flhDC*, the main operon for flagellar synthesis and thus motility (Ogasawara et al., 2011; Chambers and Sauer, 2013). In our study, RNA sequencing results revealed upregulation of *csgD, csgBAC*, and *adrA* and downregulation of *flhDC* in IMT17433 as compared to PCV17433. These results not only confirm the phenotypic data, but also emphasize the central importance of the CsgD protein in regulating the subtle interaction between bacterial sessile and planktonic ways of life.

Overall, the phenotypic tests performed for all seven strain combinations did not show a consistent pattern. Although, this is expected due to high diversity among the group of ESBL-plasmids as well as in the origins of host strains and their phylogenetic backgrounds, such inconsistencies weaken the generalizability of our conclusions. Rather, our results should be seen as an impetus for prospective studies. Interestingly, despite ESBL-plasmid diversity, each of pIMT17433, pIMT19205, and pIMT27685 carried different candidate genes, including *hha* and *icc*, which both have been described to be involved in biofilm formation and motility (Barrios et al., 2006; Kalivoda et al., 2013). Only further experiments that include cloning and knockout methods will provide more information on underlying molecular mechanisms.

In conclusion, this pilot study showed that ESBL-plasmid carriage does not necessarily lead to a growth/metabolic fitness loss. Some ESBL-plasmids in select strains may possess the potential to influence chromosomal gene expression, particularly of those genes that are important for the subtle interactions between the sessile and planktonic ways of life, such as *csgD*. We hypothesize that this may contribute to their virulence potential and pandemic success in different habitats, although underlying mechanisms remain to be identified and characterized.

## Author contributions

All authors gave their final approval of the version to be published. Conception and Design: SG, CE, KS, LW, FD. Data analysis: KS, SG, TS, DP, MD. Data acquisition: KS, SG, TS. Writing: KS, SG, LW, CE, DP, TS, MD, FD.

## Funding

This work was supported by grants from the German Research Foundation (DFG) to SG and CE entitled, “Functional analysis of non-resistance genes of extended-spectrum beta-lactamases-associated sequence types of *Escherichia coli*” (grants GU 1283/3-1 and EW116/2-1). KS was supported by a grant from SONNENFELD-Stiftung. Sequencing was partly financed by the Wellcome Trust Sanger Institute (Cambridge, United Kingdom-study number 2433ILB). The foundations that funded this work had no role in study design, data collection or interpretation, or the decision to submit the work for publication.

### Conflict of interest statement

The authors declare that the research was conducted in the absence of any commercial or financial relationships that could be construed as a potential conflict of interest.

## References

[B1] Alikhan N. F. Petty N. K. Ben Zakour N. L. Beatson S. A. (2011). BLAST Ring Image Generator (BRIG): simple prokaryote genome comparisons. BMC Genomics 12:402. 10.1186/1471-2164-12-402 21824423 PMC3163573

[B2] Anders S. Huber W. (2010). Differential expression analysis for sequence count data. Genome Biol. 11:R106. 10.1186/gb-2010-11-10-r106 20979621 PMC3218662

[B3] Andersson D. I. Hughes D. (2010). Antibiotic resistance and its cost: is it possible to reverse resistance? Nat. Rev. Microbiol. 8, 260–271. 10.1038/nrmicro2319 20208551

[B4] Barrios A. F. G. Zuo R. J. Ren D. C. Wood T. K. (2006). Hha, YbaJ, and OmpA regulate *Escherichia coli* K12 biofilm formation and conjugation plasmids abolish motility. Biotechnol. Bioeng. 93, 188–200. 10.1002/bit.20681 16317765

[B5] Blomfield I. C. Mcclain M. S. Eisenstein B. I. (1991). Type 1 fimbriae mutants of *Escherichia coli* K12: characterization of recognized afimbriate strains and construction of new fim deletion mutants. Mol. Microbiol. 5, 1439–1445. 10.1111/j.1365-2958.1991.tb00790.x 1686292

[B6] Bokranz W. Wang X. Tschape H. Romling U. (2005). Expression of cellulose and curli fimbriae by *Escherichia coli* isolated from the gastrointestinal tract. J. Med. Microbiol. 54, 1171–1182. 10.1099/jmm.0.46064-0 16278431

[B7] Bonnedahl J. Hernandez J. Stedt J. Waldenstrom J. Olsen B. Drobni M. (2014). Extended-spectrum beta-lactamases in *Escherichia coli* and *Klebsiella pneumoniae* in Gulls, Alaska, USA. Emerg. Infect. Dis. 20, 897–899. 10.3201/eid2005.130325 24750592 PMC4012786

[B8] Calhau V. Ribeiro G. Mendonca N. Da Silva G. J. (2013). Prevalent combination of virulence and plasmidic-encoded resistance in ST 131 *Escherichia coli* strains. Virulence. 4, 726–729. 10.4161/viru.26552 24128612 PMC3925704

[B9] Chambers J. R. Sauer K. (2013). Small RNAs and their role in biofilm formation. Trends Microbiol. 21, 39–49. 10.1016/j.tim.2012.10.008 23178000 PMC3752386

[B10] Chen F. Mackey A. J. Stoeckert C. J. Jr. Roos D. S. (2006). OrthoMCL-DB: querying a comprehensive multi-species collection of ortholog groups. Nucleic Acids Res. 34, D363–D368. 10.1093/nar/gkj123 16381887 PMC1347485

[B11] CLSI (2008) Performance Standards for Antimicrobial Disk and Dilution Susceptibility Tests for Bacteria Isolated from Animals; Approved Standard 3rd Edn. Wayne, PA: CLSI.

[B12] Dale J. W. Park S. F. (2004). Molecular Genetics of Bacteria. Chichester: John Wiley & Sons Ltd.

[B13] Dasilva N. A. Bailey J. E. (1986). Theoretical growth-yield estimates for recombinant cells. Biotechnol. Bioeng. 28, 741–746. 10.1002/bit.260280514 18555386

[B14] Dudin O. Geiselmann J. Ogasawara H. Ishihama A. Lacour S. (2014). Repression of flagellar genes in exponential phase by CsgD and CpxR, two crucial modulators of *Escherichia coli* biofilm formation. J. Bacteriol. 196, 707–715. 10.1128/JB.00938-13 24272779 PMC3911157

[B15] Dunn P. (2013). SPSS survival manual: a step by step guide to data analysis using IBM SPSS. Aust. Nz. J. Publ. Heal. 37, 597–598. 10.1111/1753-6405.12166

[B16] Ewers C. Bethe A. Stamm I. Grobbel M. Kopp P. A. Guerra B.. (2014). CTX-M-15-D-ST648 *Escherichia coli* from companion animals and horses: another pandemic clone combining multiresistance and extraintestinal virulence? J. Antimicrob. Chemother. 69, 1224–1230. 10.1093/jac/dkt516 24398338

[B17] Ewers C. Bethe A. Semmler T. Guenther S. Wieler L. H. (2012). Extended-spectrum beta-lactamase-producing and AmpC-producing *Escherichia coli* from livestock and companion animals, and their putative impact on public health: a global perspective. Clin. Microbiol. Infect. 18, 646–655. 10.1111/j.1469-0691.2012.03850.x 22519858

[B18] Ewers C. Grobbel M. Stamm I. Kopp P. A. Diehl I. Semmler T.. (2010). Emergence of human pandemic O25:H4-ST131 CTX-M-15 extended-spectrum beta-lactamase-producing *Escherichia coli* among companion animals. J. Antimicrob. Chemother. 65, 651–660. 10.1093/jac/dkq004 20118165

[B19] Ghigo J. M. (2001). Natural conjugative plasmids induce bacterial biofilm development. Nature 412, 442–445. 10.1038/35086581 11473319

[B20] Gophna U. Barlev M. Seijffers R. Oelschlager T. A. Hacker J. Ron E. Z. (2001). Curli fibers mediate internalization of *Escherichia coli* by eukaryotic cells. Infect. Immun. 69, 2659–2665. 10.1128/IAI.69.4.2659-2665.2001 11254632 PMC98204

[B21] Green A. M. Sambrook J. (2012). Molecular Cloning: A Labarotory Manual. Cold Springer Harbor, NY: Cold Springer Harbor Labarotory Press.

[B22] Hammar M. Arnqvist A. Bian Z. Olsen A. Normark S. (1995). Expression of two csg operons is required for production of fibronectin- and congo red-binding curli polymers in *Escherichia coli* K-12. Mol. Microbiol. 18, 661–670. 10.1111/j.1365-2958.1995.mmi_18040661.x 8817489

[B23] Harshey R. M. (2003). Bacterial motility on a surface: many ways to a common goal. Annu. Rev. Microbiol. 57, 249–273. 10.1146/annurev.micro.57.030502.091014 14527279

[B24] Hayashi K. Morooka N. Yamamoto Y. Fujita K. Isono K. Choi S.. (2006). Highly accurate genome sequences of *Escherichia coli* K-12 strains MG1655 and W3110. Mol. Syst. Biol. 2, 2006.0007. 10.1038/msb4100049 16738553 PMC1681481

[B25] Ito A. Taniuchi A. May T. Kawata K. Okabe S. (2009). Increased antibiotic resistance of *Escherichia coli* in mature biofilms. Appl. Environ. Microbiol. 75, 4093–4100. 10.1128/AEM.02949-08 19376922 PMC2698376

[B26] Joensen K. G. Scheutz F. Lund O. Hasman H. Kaas R. S. Nielsen E. M.. (2014). Real-time whole-genome sequencing for routine typing, surveillance, and outbreak detection of verotoxigenic *Escherichia coli*. J. Clin. Microbiol. 52, 1501–1510. 10.1128/JCM.03617-13 24574290 PMC3993690

[B27] Johnson J. R. Johnston B. Clabots C. Kuskowski M. A. Castanheira M. (2010). *Escherichia coli* sequence type ST131 as the major cause of serious multidrug-resistant *E. coli* infections in the United States. Clin. Infect. Dis. 51, 286–294. 10.1086/653932 20572763

[B28] Kalivoda E. J. Brothers K. M. Stella N. A. Schmitt M. J. Shanks R. M. Q. (2013). Bacterial cyclic AMP-phosphodiesterase activity coordinates biofilm formation. PLoS ONE 8:e71267. 10.1371/journal.pone.0071267 23923059 PMC3726613

[B29] Kearse M. Moir R. Wilson A. Stones-Havas S. Cheung M. Sturrock S.. (2012). Geneious basic: an integrated and extendable desktop software platform for the organization and analysis of sequence data. Bioinformatics 28, 1647–1649. 10.1093/bioinformatics/bts199 22543367 PMC3371832

[B30] Kim D. Pertea G. Trapnell C. Pimentel H. Kelley R. Salzberg S. L. (2013). TopHat2: accurate alignment of transcriptomes in the presence of insertions, deletions and gene fusions. Genome Biol. 14:R36. 10.1186/gb-2013-14-4-r36 23618408 PMC4053844

[B31] Lanza V. F. De Toro M. Garcillan-Barcia M. P. Mora A. Blanco J. Coque T. M.. (2014). Plasmid Flux in *Escherichia coli* ST131 sublineages, analyzed by plasmid constellation network (PLACNET), a new method for plasmid reconstruction from whole genome sequences. PLoS Genet. 10:e1004766. 10.1371/journal.pgen.1004766 25522143 PMC4270462

[B32] La Ragione R. M. Collighan R. J. Woodward M. J. (1999). Non-curliation of *Escherichia coli* O78:K80 isolates associated with IS1 insertion in csgB and reduced persistence in poultry infection. FEMS Microbiol. Lett. 175, 247–253. 10.1111/j.1574-6968.1999.tb13627.x 10386375

[B33] Lehoux I. E. Mazzulla M. J. Baker A. Petit C. M. (2003). Purification and characterization of YihA, an essential GTP-binding protein from *Escherichia coli*. Protein Expr. Purif. 30, 203–209. 10.1016/S1046-5928(03)00107-4 12880769

[B34] Lenski R. E. (1997). The cost of antibiotic resistance - from the perspective of a bacterium. antibiotic resistance: origins, evolution, selection and spread. CIBA Found. Symp. 207, 131–140. 9189639 10.1002/9780470515358.ch9

[B35] Li B. Dewey C. N. (2011). RSEM: accurate transcript quantification from RNA-Seq data with or without a reference genome. BMC Bioinformatics 12:323. 10.1186/1471-2105-12-323 21816040 PMC3163565

[B36] Martinez-Medina M. Naves P. Blanco J. Aldeguer X. Blanco J. E. Blanco M.. (2009). Biofilm formation as a novel phenotypic feature of adherent-invasive *Escherichia coli* (AIEC). BMC Microbiol. 9:202. 10.1186/1471-2180-9-202 19772580 PMC2759958

[B37] May T. Okabe S. (2008). *Escherichia coli* harboring a natural IncF conjugative F plasmid develops complex mature biofilms by stimulating synthesis of colanic acid and Curli. J. Bacteriol. 190, 7479–7490. 10.1128/JB.00823-08 18790864 PMC2576669

[B38] Naseer U. Sundsfjord A. (2011). The CTX-M Conundrum: dissemination of plasmids and *Escherichia coli* clones. Microb. Drug Resist. 17, 83–97. 10.1089/mdr.2010.0132 21281129

[B39] Nicolas-Chanoine M. H. Bertrand X. Madec J. Y. (2014). *Escherichia coli* ST131, an intriguing clonal group. Clin. Microbiol. Rev. 27, 543–574. 10.1128/CMR.00125-13 24982321 PMC4135899

[B40] Nicolas-Chanoine M. H. Blanco J. Leflon-Guibout V. Demarty R. Alonso M. P. Canica M. M.. (2008). Intercontinental emergence of *Escherichia coli* clone O25:H4-ST131 producing CTX-M-15. J. Antimicrob. Chemother. 61, 273–281. 10.1093/jac/dkm464 18077311

[B41] Ogasawara H. Yamamoto K. Ishihama A. (2011). Role of the biofilm master regulator CsgD in cross-regulation between biofilm formation and flagellar synthesis. J. Bacteriol. 193, 2587–2597. 10.1128/JB.01468-10 21421764 PMC3133154

[B42] Pardee A. B. Jacob F. Monod J. (1959). Genetic control and cytoplasmic expression of inducibility in the synthesis of beta-galactosidase by *E. Coli*. J. Mol. Biol. 1, 165–178. 10.1016/S0022-2836(59)80045-0

[B43] Perez-Rueda E. Collado-Vides J. (2000). The repertoire of DNA-binding transcriptional regulators in *Escherichia coli* K-12. Nucleic. Acids. Res. 28, 1838–1847. 10.1093/nar/28.8.1838 10734204 PMC102813

[B44] Pitout J. D. (2012). Extraintestinal pathogenic *Escherichia coli*: a combination of virulence with antibiotic resistance. Front. Microbiol. 3:9. 10.3389/fmicb.2012.00009 22294983 PMC3261549

[B45] Richter A. (2011). Wachstumsverhalten, Motilität und Biofilm-Bildung der Escherichia coli K-12 Laborstämme W3110, MG1655 und MC4100. Ph.D. Thesis, Humboldt Universität Berlin.

[B46] Richter A. M. Povolotsky T. L. Wieler L. H. Hengge R. (2014). Cyclic-di-GMP signalling and biofilm-related properties of the Shiga toxin-producing 2011 German outbreak *Escherichia coli* O104:H4. EMBO Mol. Med. 6, 1622–1637. 10.15252/emmm.201404309 25361688 PMC4287979

[B47] Robinson M. D. Mccarthy D. J. Smyth G. K. (2010). edgeR: a Bioconductor package for differential expression analysis of digital gene expression data. Bioinformatics 26, 139–140. 10.1093/bioinformatics/btp616 19910308 PMC2796818

[B48] Romling U. (2005). Characterization of the rdar morphotype, a multicellular behaviour in Enterobacteriaceae. Cell. Mol. Life Sci. 62, 1234–1246. 10.1007/s00018-005-4557-x 15818467 PMC11139082

[B49] Ross P. Mayer R. Benziman M. (1991). Cellulose biosynthesis and function in bacteria. Microbiol. Rev. 55, 35–58. 2030672 10.1128/mr.55.1.35-58.1991 PMC372800

[B50] Sahly H. Navon-Venezia S. Roesler L. Hay A. Carmeli Y. Podschun R.. (2008). Extended-spectrum beta-lactamase production is associated with an increase in cell invasion and expression of fimbrial adhesins in *Klebsiella pneumoniae*. Antimicrob. Agents Chemother. 52, 3029–3034. 10.1128/AAC.00010-08 18573929 PMC2533491

[B51] Schaufler K. Wieler L. H. Semmler T. Ewers C. Guenther S. (2013). ESBL-plasmids carrying toxin-antitoxin systems can be “cured” of wild-type *Escherichia coli* using a heat technique. Gut. Pathog. 5:34. 10.1186/1757-4749-5-34 24245987 PMC4177129

[B52] Schierack P. Romer A. Jores J. Kaspar H. Guenther S. Filter M.. (2009). Isolation and characterization of intestinal *Escherichia coli* clones from wild boars in Germany. Appl. Environ. Microbiol. 75, 695–702. 10.1128/AEM.01650-08 19060173 PMC2632144

[B53] Schmieder R. Lim Y. W. Edwards R. (2012). Identification and removal of ribosomal RNA sequences from metatranscriptomes. Bioinformatics. 28, 433–435. 10.1093/bioinformatics/btr669 22155869 PMC3268242

[B54] Seiler C. Berendonk T. U. (2012). Heavy metal driven co-selection of antibiotic resistance in soil and water bodies impacted by agriculture and aquaculture. Front. Microbiol. 3:399. 10.3389/fmicb.2012.00399 23248620 PMC3522115

[B55] Smet A. Martel A. Persoons D. Dewulf J. Heyndrickx M. Claeys G.. (2010a). Characterization of extended-spectrum beta-lactamases produced by *Escherichia coli* isolated from hospitalized and nonhospitalized patients: emergence of CTX-M-15-producing strains causing urinary tract infections. Microb. Drug Res. 16, 129–134. 10.1089/mdr.2009.0132 20370505

[B56] Smet A. Van Nieuwerburgh F. Vandekerckhove T. T. M. Martel A. Deforce D. Butaye P.. (2010b). Complete nucleotide sequence of CTX-M-15-Plasmids from clinical *Escherichia coli* isolates: insertional events of transposons and insertion sequences. PLoS ONE 5:e11202. 10.1371/journal.pone.0011202 20585456 PMC2887853

[B57] Smirnov N. (1948). Table for estimating the goodness of fit of empirical distributions. Ann. Math. Stat. 19, 279–279. 10.1214/aoms/1177730256

[B58] Stead M. B. Agrawal A. Bowden K. E. Nasir R. Mohanty B. K. Meagher R. B.. (2012). RNAsnap (TM): a rapid, quantitative and inexpensive, method for isolating total RNA from bacteria. Nucleic Acids Res. 40, e156. 10.1093/nar/gks680 22821568 PMC3488207

[B59] Trapnell C. Hendrickson D. G. Sauvageau M. Goff L. Rinn J. L. Pachter L. (2013). Differential analysis of gene regulation at transcript resolution with RNA-seq. Nat. Biotechnol. 31, 46–53. 10.1038/nbt.2450 23222703 PMC3869392

[B60] Vaas L. A. Sikorski J. Hofner B. Fiebig A. Buddruhs N. Klenk H. P.. (2013). opm: an R package for analysing OmniLog^(*R*)^ phenotype microarray data. Bioinformatics 29, 1823–1824. 10.1093/bioinformatics/btt291 23740744

[B61] Vaas L. A. Sikorski J. Michael V. Goker M. Klenk H. P. (2012). Visualization and curve-parameter estimation strategies for efficient exploration of phenotype microarray kinetics. PLoS ONE 7:e34846. 10.1371/journal.pone.0034846 22536335 PMC3334903

[B62] Von Mentzer A. Connor T. R. Wieler L. H. Semmler T. Iguchi A. Thomson N. R.. (2014). Identification of enterotoxigenic *Escherichia coli* (ETEC) clades with long-term global distribution. Nat. Genet. 46, 1321–1326. 10.1038/ng.3145 25383970

[B63] Wilcoxon F. (1945). Individual comparisons by ranking methods. Biometrics Bull. 1, 80–83. 10.2307/3001968

[B64] Woodford N. Turton J. F. Livermore D. M. (2011). Multiresistant Gram-negative bacteria: the role of high-risk clones in the dissemination of antibiotic resistance. FEMS Microbiol. Rev. 35, 736–755. 10.1111/j.1574-6976.2011.00268.x 21303394

[B65] Yang X. Ma Q. Wood T. K. (2008). The R1 conjugative plasmid increases *Escherichia coli* biofilm formation through an envelope stress response. Appl. Environ. Microbiol. 74, 2690–2699. 10.1128/AEM.02809-07 18344336 PMC2394867

[B66] Zankari E. Hasman H. Cosentino S. Vestergaard M. Rasmussen S. Lund O.. (2012). Identification of acquired antimicrobial resistance genes. J. Antimicrob. Chemother. 67, 2640–2644. 10.1093/jac/dks261 22782487 PMC3468078

[B67] Zhao K. Liu M. Burgess R. R. (2007). Adaptation in bacterial flagellar and motility systems: from regulon members to ‘foraging’-like behavior in *E. coli*. Nucleic Acids Res. 35, 4441–4452. 10.1093/nar/gkm456 17576668 PMC1935009

[B68] Zogaj X. Nimtz M. Rohde M. Bokranz W. Romling U. (2001). The multicellular morphotypes of *Salmonella typhimurium* and *Escherichia coli* produce cellulose as the second component of the extracellular matrix. Mol. Microbiol. 39, 1452–1463. 10.1046/j.1365-2958.2001.02337.x 11260463

