# Supplementary material for: Carriage of Extended-Spectrum Beta-Lactamase-Plasmids Does Not Reduce Fitness but Enhances Virulence in Some Strains of Pandemic E. coli Lineages
Source: Front Microbiol. 2016 Mar 17;7:336. doi: 10.3389/fmicb.2016.00336 (PMC4794485; doi:10.3389/fmicb.2016.00336)
Supplement: Supplementary file 1 [file DataSheet1.DOCX]

| **Strain** | **ST** | **Host** | **Origin** | **ESBL type** | **Growth curves** | **Omnilog®** | **Macrocolonies** | **Biofilm** | **Motility** | **RNA Seq.** |
| --- | --- | --- | --- | --- | --- | --- | --- | --- | --- | --- |
| IMT17433* | 131 | Dog *(C. lupus familiaris)* | UTI | Wild-type CTX-M-15 | ND | SD | SD | SD | SD | SD |
| PCV17433 | 131 |  |  | ESBL-plasmid-"cured" | ND | SD | SD | SD | SD | SD |
| T17433 | 131 |  |  | Transformed CTX-M-15 | NT | R | R | NR | R | R |
| IMT19205 | 131 | Brown rat *(R. norvegicus)* | Feces | Wild-type CTX-M-27 | ND | SD | ND | NSD | NSD | NT |
| PCV19205 | 131 |  |  | ESBL-plasmid-"cured" | ND | SD | ND | NSD | NSD | NT |
| T19205 | 131 |  |  | Transformed CTX-M-27 | NT | R | ND | NSD | NSD | NT |
| IMT27685 | 131 | Raven *(C. corax)* | Feces | Wild-type CTX-M-15 | ND | SD | ND | SD | NSD | NT |
| PCV27685 | 131 |  |  | ESBL-plasmid-"cured" | ND | SD | ND | SD | NSD | NT |
| T27685 | 131 |  |  | Transformed CTX-M-15 | NT | R | ND | NR | NSD | NT |
| IMT16316 | 648 | Blackbird *(T. merula)* | Feces | Wild-type CTX-M-15 | ND | SD | SD | NSD | NSD | NT |
| PCV16316 | 648 |  |  | ESBL-plasmid-"cured" | ND | SD | SD | NSD | NSD | NT |
| T16316 | 648 |  |  | Transformed CTX-M-15 | NT | R | R | NSD | NSD | NT |
| IMT17887** | 648 | Horse *(E. ferus caballus)* | WI | Wild-type CTX-M-15 | ND | SD | SD | SD | SD | NT |
| PCV17887 | 648 |  |  | ESBL-plasmid-"cured" | ND | SD | SD | SD | SD | NT |
| T17887 | 648 |  |  | Transformed CTX-M-15 | NT | R | R | NR | R | NT |
| IMT21183 | 648 | Human *(H. sapiens)* | UTI | Wild-type CTX-M-15 | ND | SD | ND | NSD | NSD | NT |
| PCV21183 | 648 |  |  | ESBL-plasmid-"cured" | ND | SD | ND | NSD | NSD | NT |
| T21183 | 648 |  |  | Transformed CTX-M-15 | NT | R | ND | NSD | NSD | NT |
| IMT23463 | 648 | Monk vulture *(A. monachus)* | Feces | Wild-type CTX-M-14 | ND | SD | ND | NSD | NSD | NT |
| PCV23463 | 648 |  |  | ESBL-plasmid-"cured" | ND | SD | ND | NSD | NSD | NT |
| T23463 | 648 |  |  | Transformed CTX-M-14 | NT | R | ND | NSD | NSD | NT |

Supplementary table S1: Overview of designation, origin, features and results of tested *E. coli* strains (Abbreviations: */**: strains provided by IDEXX Vet. Med. Lab., Ludwigsburg, Germany, original designation: *VB964041.2, **VB977549, IMT: Institut für Mikrobiologie und Tierseuchen, PCV: ESBL-plasmid-“cured” variant, T: transformant, ST: sequence type, UTI: urinary tract infection, WI: wound infection, ND: no difference between wild-type and ESBL-plasmid-“cured” variant [in all runs], NT: not tested, SD: significant difference between wild-type and ESBL-plasmid-“cured” variant [in most runs], R: reversibility in transformants; meaning that phenotype/transcriptome was similar to the corresponding wild-type strain [in most runs], NSD: no significant difference between wild-type and ESBL-plasmid-“cured” variant [in all runs], NR: no reversibility in transformants; meaning that phenotype/transcriptome was not similar to the corresponding wild-type strain [in all runs]).
