# Supplementary material for: Carriage of Extended-Spectrum Beta-Lactamase-Plasmids Does Not Reduce Fitness but Enhances Virulence in Some Strains of Pandemic E. coli Lineages
Source: Front Microbiol. 2016 Mar 17;7:336. doi: 10.3389/fmicb.2016.00336 (PMC4794485; doi:10.3389/fmicb.2016.00336)
Supplement: Supplementary file 2 [file DataSheet2.DOCX]

| **Strain** | **PM1, Wells** | **PM2, Wells** | **PM3, Wells** | **PM4, Wells** | **PM13, Wells** | **PM14, Wells** |
| --- | --- | --- | --- | --- | --- | --- |
| IMT17433 | F7 |  | A7, B1, B2, B9, B10, C3, C8, E8, E12, G12, H1, H4, H7, H10 | A10, B3, B5, B7, B8, B9, B10, C3, C4, C6, C8, C9, C10, C11, D2, D9, D10, D11 | C6, C7, D4, D11 | G6, G7, G8 |
| PCV17433 | A8, B12 | E8 | B4, B5, C9, C11, D2, G8 | A12, C5, E6, G6 |  |  |
| T17433 | F7 |  | A7, B1, B2, B9, B10, C3, C8, E8, E12, G12, H1, H4, H7, H10 | A10, B3, B5, B7, B8, B9, B10, C3, C4, C6, C8, C9, C10, C11, D2, D9, D10, D11 | C6, C7, D4, D11 | G6, G7, G8 |
| IMT19205 |  |  |  |  | D4 |  |
| PCV19205 |  |  |  |  |  |  |
| T19205 |  |  |  |  | D4 |  |
| IMT27685 |  |  |  |  | C6, D4 | G6, G7, G8 |
| PCV27685 |  |  |  |  |  |  |
| T27685 |  |  |  |  | C6, D4 | G6, G7, G8 |
| IMT16316 |  |  |  |  | C6, C7, D4, D12 | G8 |
| PCV16316 |  | B3 |  |  |  |  |
| T16316 |  |  |  |  | C6, C7, D4, D12 | G8 |
| IMT17887 |  |  | E12 |  | C6, C7, D12 | G6, G7, G8 |
| PCV17887 | A10, C9, G4 | B3 |  | A4, A8, B2, D8, E6 |  |  |
| T17887 |  |  | E12 |  | C6, C7, D12 | G6, G7, G8 |
| IMT21183 |  |  |  |  | B4, C6, C7, D4 | G7 |
| PCV21183 |  |  |  |  |  |  |
| T21183 |  |  |  |  | B4, C6, C7, D4 | G7 |
| IMT23463 |  |  |  |  |  | G6, G7, G8 |
| PCV23463 |  |  |  |  |  |  |
| T23463 |  |  |  |  |  | G6, G7, G8 |

Supplementary table S2: Results of Omnilog® phenotype microarray (PM) assays of tested strains on six different PM plates. Plates were incubated at 37°C for 48 hours. Significant differences are indicated in the table per single well (Substrates in wells: PM1: A8: L-Proline, A10: D-Trehalose, B12: L-Glutamic Acid, C9: D-Glucose, F7: Propionic Acid, G4: L-Threonine; PM2: B3: beta-D-Allose, E8: beta-Hydroxy Butyric Acid; PM3: A7: L-Alanine, B1: L-Glutamine, B2: Glycine, B4: L-Isoleucine, B5: L-Leucine, B9: L-Proline, B10: L-Serine, C3: D-Alanine, C8: D-Serine, C9: D-Valine, C11: L-Homoserine, D2: N-Phthaloyl-L-glutamic acid, E8: D-Glucosamine, E12: N-Acetyl-D-Galactosamine, F1: N-Acetyl-D-Mannosamine, G8: gamma-Amino-N-Butyric Acid, G12: alpha-Amino-N-Valeric Acid, H1: Ala-Asp, H4: Ala-Gly, H7: Ala-Thr, H10: Gly-Glu; PM4: A4: Trimetaphosphate, A8: Adenosine- 2’-Monophosphate, A10: L-Aspartic Acid, A12: L-Glutamic Acid, B2: Dithiophosphate, B3: L-Histidine, B5: L-Leucin, B7: L-Methionine, B8: L-Phenylalanine, B9: L-Proline, B10: L-Serine, C3: D-Alanine, C5: 2-Desoxy-D-Glucose-6-phosphate, C4: D-Asparagine, C6: D-Glutamic Acid, C8: D-Serine, C9: D-Valine, C10: L-Citrulline, C11: L-Homoserine, D2: N-Phthaloyl-L-glutamic Acid, D8: Uridine- 2’-Monophosphate, D9: Ethanolamine, D10: Ethylenediamine, D11: Putrescine, E6: Phosphono Acetic Acid, G6: D,L-Ethionine; PM13: B4: Azlocillin, C6, C7: Doxycyclin, D4: Cefuroxime, D11, D12: Rolitetracyclin; PM14: G6, G7, G8: Carbenicillin).
